# Propolis: A New Alternative for Root Canal Disinfection

**Published:** 2012-08-01

**Authors:** Maryam Zare Jahromi, Hasan Toubayani, Majid Rezaei

**Affiliations:** 1. Department of Dentistry, Khorasgan Branch, Islamic Azad University, Isfahan, Iran; 2. Dentist, Isfahan, Iran; 3. Department of Oral and Maxillofacial Surgery, Isfahan University of medical sciences, Isfahan, Iran

**Keywords:** Calcium Hydroxide, Disinfection, Enterococcus Faecalis, Propolis, Root Canal

## Abstract

**Introduction:**

This study evaluated and compared colony forming units (CFUs) and minimum inhibitory concentrations (MICs) of calcium hydroxide and propolis as intracanal medicaments.

**Materials and Methods:**

Eighty human single-root and caries-free teeth were selected and divided into five groups. Crowns were removed. Root canals were then prepared in a step-back manner. The samples were then inoculated by Enterococcus (E.) faecalis and incubated for 21 days. Intracanal medications were applied including, calcium hydroxide (n=20), propolis (n=20), and ethanol (n=20). Two groups of 10 teeth were also used as the positive and negative controls. Microbiological sampling was performed utilizing a piezo-reamer drill after one week of incubation. The samples were plated and CFUs were counted after 48 hours. MICs of calcium hydroxide and propolis were measured by serial dilution and agar dilution methods, respectively. The statistical tests of ANOVA and Duncan post-hoc were used to compare different medications.

**Results:**

MICs and CFUs of propolis were dramatically less than calcium hydroxide. The difference between the groups was statistically significant (P<0.001).

**Conclusion:**

Our results reveal that propolis is an effective antimicrobial intracanal agent.

## Introduction

Elimination or reduction of microorganisms in the root canal system has been the principal idea in endodontics since the basic work of Kakehashi et al. [1]; however, few root canals have shown to be bacteria-free following mechanical instrumentation [2]). Intracanal antimicrobial agents as an adjunct therapy have been the next step in endodontics. Phenolic compounds such as eugenol and camphorated monochlorophenol (CMCP), aldehydes such as formocresol, and halides such as iodine potassium iodide are examples of different chemical compounds that have been used for decades [3]. These medicaments are not recommended due to antigenic and cytotoxic properties [3][4][5][6][7]. Of all medicaments discussed in the literature, calcium hydroxide Ca(OH)_2_ is the most commonly used and the most effective [8][9][10]. It was first introduced by Hermann in 1920 [11] and many indications have been found since. Apexification, apexogenesis, root perforation, and fracture are some procedures in which Ca(OH)_2_ is used. It is also applied as an inter-appointment intracanal dressing due to its bactericidal activity. Its high pH is thought to provide Ca(OH)_2_ with antimicrobial property [12][13][14]. However, Ca(OH)_2_ is not able to eradicate some bacterial species like Enterococcus (E.) faecalis, the main microorganism found in root canal therapy failures [15][16]. E. faecalis is an opportunistic gram-positive cocci whose resistance to a wide range of antibiotics has made it a problematic nosocomial pathogen [17][18]. Although E. faecalis is rarely isolated from the primary endodontic infections, it is frequently associated with endodontic flare-ups. Rocas et al. in their study on 30 endodontically treated teeth with persistent periapical infection demonstrated that 20 lesions were associated with E. faecalis [19]. Considering the shortcomings of Ca(OH)_2_ in eradication of E. faecalis, some have focused on alternative medicaments with greater bactericidal potency. Of the newly found medications, propolis has attracted attention as a natural antimicrobial agent. Propolis (bee glue) is a byproduct of honeybees that is widely used in traditional medicine. Global trends toward natural products have been the stimulus for further investigation of medical potentials of propolis.

It is well documented that propolis has antibacterial, antiviral, and antifungal properties [20][21][22]. In an in vitro study, Oncag et al. showed that different types of propolis had antimicrobial activity against E. faecalis [23]. Results of another study by Stepanovic et al. revealed synergistic action of propolis with antibiotics [24]. Propolis properties depend upon it’s chemical ingredients . Location, season, and vegetation of the area from which propolis is collected influence its composition and biological activity [25]. The aim of this study was to compare the antibacterial potency of propolis with Ca(OH)_2_. To the best of our knowledge, it is the third study in which the intracanal environment is simulated.

## Materials and methods

Eighty freshly extracted human single-rooted teeth with straight canals and well-developed apices were selected for this study. All teeth were caries-free and had been extracted for periodontal or orthodontic reasons. In order to remove debris and perform initial disinfection, the teeth were dipped in 5.25% NaOCl for 30 minutes and then were rinsed with distilled water. To standardize root canal preparation, crowns of all teeth were cut 14 mm away from the apex by the means of a non-stop diamond disc. Thereafter, root canals were prepared in a step-back fashion (with a working length of 12 mm) with K-files up to #50 followed by piezoreamer no. 1, 2, and 3. Instrumentation with each rotary bur was carried out at a given speed and time (3600 rpm/2 sec) to achieve uniform and isovolumic root canals. At the end, the root canals were instrumented 0.5 mm beyond the apex by a small K-file to confirm the apical patency. After the preparation was complete, the apical region was sealed by flowable composite resin (Esthet-X, Dentsply, York, PA, USA) to block bacterial microleakage. The smear layer was then removed in an ultrasonic bath containing 17% EDTA for 4 minutes followed by 5.25% NaOCl for another 4 minutes. The teeth were then rinsed by 10 mL of physiologic saline and autoclaved twice at 121ºC for 30 minutes. To provide an enriched environment in dentinal tubules for bacterial growth, Brain Heart Infusion (BHI) culture medium (Pronadisa, Madrid, Spain) was used in an ultrasonic bath (5 min) for better penetration of the medium into the dentin. Thereafter, the samples were incubated in 37ºC for a week. Two control teeth were plated in BHI to confirm the sterility of the samples. Following this stage, the samples were dipped in BHI liquid medium containing E. faecalis (ATCC 51299) and adjusted spectrophotometrically to 0.5 McFarland standard (1.5×108 bacteria/mL). The samples in culture medium were kept in an incubator at 37ºC for 21 days. Bacteria-infected culture medium was substituted with a fresh one every 3 days.

### Preparation of Propolis

Propolis samples were obtained from the beehives of Najaf Abad, Esfahan. Three-hundred grams of frozen propolis was ground and dissolved in 300ml 96% ethanol at 37ºC to obtain 100% (w/v) extract. The mixture was poured into a bottle and incubated at 30ºC for 2 weeks. After incubation, the supernatant mixture was filtered twice with Whatman no. 4 and 1 filter paper. The filtered mixture (Eppendorf, Hamburg, Germany) was concentrated at 30ºC for 6 hours (1500 rpm). The final extraction of propolis obtained a density of 150 mg/mL.

### Intracanal Medications

After 3 weeks, the infected samples were divided into five groups. Antimicrobial medicaments were injected into the 40 experimental canals until they were filled (Group I, II). Group IV received no medicament in order to demonstrate a suitable environment for bacterial growth (positive control). Group V consisted of the sterile samples, to indicate sterility of the procedure (negative control). Once the injection was complete, the orifices of the canals were sealed with Zonalin (Kemdent, Wiltshire, UK). Alcohol content in group I and III was allowed to evaporate before the application of the Zonalin. All surfaces of each sample were additionally sealed with two layers of nail varnish. The samples were then wrapped in sterile saline-soaked gauze to prevent dehydration of the teeth. Finally, they were sealed in a plate and incubated at 37ºC for one week.

### Microbiological Sampling and Culture

The orifices were re-opened upon completion of the incubation and canals were rinsed with sterile normal saline. To confirm complete removal of intracanal medicament, a sterile file was used while rinsing. The canals were finally dried by means of a sterile paper point. Piezo-reamer no. 4 (3500 rpm) was applied up to the working length for about 2 seconds in order to obtain dentinal chips. The drills were then collected into test tubes containing BHI. Serial 10-fold dilutions were made by the means of BHI broth as the diluent. From the serial dilutions, 0.1ml was transferred and plated on Mueller-Hinton agar (Merck, Darmstadt, Germany). After one week of incubation at 37ºC, colony-forming units (CFU) were counted.

### Minimum Inhibitory Concentration (MIC)

We followed the guidelines of National Committee for Clinical Laboratory Standards (NCCLS) to determine the MIC of different medicaments. A serial dilution method was performed for Ca(OH)_2_ and ethanol; however, we applied an agar dilution method for propolis.

Serial dilution of the nine test tubes (culture medium plus 5×10^5^ bacteria), was performed by adding different concentrations (1000, 500, 250, 125, 62.5, 31.2, 15.6, 7.8, and 3.9 µg/mL) of 1% Ca(OH)_2_ (Merck, Darmstadt, Germany). The negative control test tube did not receive any bacterial suspension. Another test tube (positive control) contained bacterial suspension without any medication to show the capability of bacterial growth and production of complete haziness. The series of 11 tubes was incubated at 37ºC for 24 hours. The same procedure was performed for 96% ethanol. The serial dilution included 96, 48, 24, 12, 6, 3, and 1.5%. For both medicaments, the last tube which was completely clear (no haziness) was designated as MIC.

### Agar dilution

Different concentrations of propolis in addition to BHI broth were added to sterile Mueller-Hinton agar at a temperature of 50ºC, mixed and poured into 16 sterile petri-plates and allowed to cool. A suspension of 5×10^5^ bacteria was spread over the plate in all directions with a standard loop. After inoculation, all plates were incubated at 37ºC for 24 hours. The lowest concentration of propolis that inhibited visible growth of bacterial spots over the plate was defined as the MIC.

All assays were performed in duplicate. The data analysis was performed using SPSS 13 (SPSS Inc., Chicago, IL, USA) statistical package. The statistical tests of ANOVA and Duncan post-hoc were used to compare different medicaments.

## Results

The results of CFU are shown in Table 1.

**Table 1:** Colony Forming Units (CFU) of different medicaments

****	**Propolis**	**Ca(OH)_2_**	**Ethanol**	**Negative Control**	**Positive Control**
1	0	102	600	0	UC a
2	20	317	450	0	UC
3	0	103	371	0	UC
4	0	201	250	2	UC
5	8	97	1000	0	UC
6	4	205	987	1	UC
7	5	99	881	0	UC
8	0	167	634	2	UC
9	21	399	432	1	UC
10	7	123	560	0	UC
11	0	88	□	□	□
12	0	97	□	□	□
13	5	160	□	□	□
14	0	171	□	□	□
15	10	207	□	□	□
16	0	82	□	□	□
17	14	200	□	□	□
18	19	300	□	□	□
19	0	295	□	□	□
20	3	258	□	□	□
Mean (SD)	5.80 (7.30)	183.55 (90.81)	616.50 (261.28)	negligible	□

^a^ UN: Uncountable

The difference between propolis, Ca(OH)_2_ and ethanol groups was statistically significant (P<0.001). A significant difference was found between medicament groups (I and II) and the ethanol group. Since ethanol, as the solvent of propolis, is considered as an antimicrobial agent, a post-hoc Duncan test further differentiates the effect of propolis and ethanol. The greatest difference was seen between propolis and ethanol. Propolis demonstrated a far lower number of CFU than Ca(OH)_2_. An uncountable amount of colonies was formed in the positive control group (ten inoculated plates without any medicament), which indicates favorable conditions for bacterial growth. In the negative control group, 1-2 colonies, if any, were present over some plates, which confirm aseptic procedures during the assays.

Minimum Inhibitory Concentrations (MICs) of propolis was 340 and MIC of Ca(OH)_2_ was 2500. Propolis showed more potency than Ca(OH)_2_, and required a much lower concentration of propolis for inhibitory activity against E. faecalis.

## Discussion

The role of bacteria in periapical pathological lesions has well been described. Contemporary endodontics therefore deals with prevention or eradication of root canal infections. Current concepts of root canal therapy advocate the combination of chemical and mechanical cleansing of the root dentin. Mechanical instrumentation has been standardized, at least in part, over recent decades; however, application of intracanal medication has been controversial.

The aim of this study was to compare propolis, a natural antimicrobial agent, to Ca(OH)_2_. E. faecalis was selected for this study since it has been a challenging and hard-to-overcome organism in the realm of periapical infections. Ca(OH)_2_ showed a moderate antimicrobial efficacy against E. faecalis in our study. Different investigations have demonstrated diverse results. Safavi et al. in 1990 showed that E. faecalis survived for a relatively long time in presence of Ca(OH)_2_ [26]. A further study by Siqueira et al. demonstrated inefficacy of Ca(OH)_2_ against E. faecalis even after one week [27]. Baker et al. in 2004 concluded that as a 24-hour medicament, Ca(OH)_2_ consistently failed to eliminate E. faecalis [28][29]. In a study conducted by Basrani et al., the authors found that Ca(OH)_2_ alone had no effect on E. faecalis [30][31]. Ca(OH)_2_ has shown no antimicrobial action after 72 hours as reported by Neelakantan et al. [32]. On the other hand, Sjogren et al. reported that a 7-day application of Ca(OH)_2_ efficiently eliminated bacteria that had survived biomechanical instrumentation of the canal, while the 10-minute application was ineffective [33][34]. Studies have shown that infection persisted in only 26% of septic canals [35][36]. Antimicrobial property of propolis has been documented. We found that propolis had significant efficacy in killing E. faecalis. In an in vitro study, Oncag et al. demonstrated that propolis had significant antimicrobial activity against E faecalis and suggested propolis to be used in endodontics [23]. Stepanovic et al. is also another proponent of propolis as an anti-E. faecalis agent [24]. A study compared two samples of propolis and showed that although propolis had some antimicrobial activity, it did not have any activity against E. faecalis [37]. E. faecalis has been shown to be moderately susceptible to propolis, in contrast to Actinomyces Viscous, which was far more susceptible [38].

In the present study, we compared antibacterial activity of propolis and Ca(OH)_2_ as intracanal medicaments. To the best of our knowledge it is the third study in which intracanal environment had been simulated. A study in 2006 showed that Ca(OH)_2_-containing medicaments worked very efficiently within the first 48-hours; however they were not as efficient after 10 days [39]. Propolis was the most effective agent after 10 days in their study.

Our results revealed that after one week of incubation, propolis was much more effective than Ca(OH)_2_, similar to 10-day results of the previous study. In contrast to Oncag’s study, another study found propolis to be significantly more effective than Ca(OH)_2_ after short-term application [40]. Although our samples were studied after one week, the results concur with their study. Microbiological sampling in Oncag’s study was performed utilizing paper points, but we used a piezo reamer as the sampler. We found an MIC of 2500 µg/mL for 1% Ca(OH)_2_. Higher concentration of 10% Ca(OH)_2_ solution was shown to produce a MIC of 1562 µg/mL [41]. The difference may be explained by different types of E. faecalis used in two studies. E. faecalis (ATCC 29212) is the strand of choice in most studies. However, this type was not available in the country at the time of the study, and so we used E. faecalis (ATCC 51299). This type is vancomycin-resistant and appears to be less susceptible to antimicrobials than ATCC 29212. MIC of propolis in our study was as low as 340 µg/mL; however Ferreira and colleagues found it to be as high as 6425 µg/mL. This difference is perhaps due to different microbiological assessment. We used an agar dilution method in contrast to Ferreira et al.’s study in which serial dilution was applied. Other researchers have applied a serial dilution method to determine MIC for different types of propolis [42]. Mean values of MIC against various pathogens were between 80 and 261 µg/mL; these values are relatively close to our measures. The MIC of propolis was 125-250 µg/mL for various gram-positive bacteria in another study [43].

It is imperative to remember that propolis of different origins have different compositions and antimicrobial activities. The composition of propolis is highly variable and its original plant, like other medicinal plants, requires standardization [44]. The diverse values of MIC in the literature may be attributed to this fact. A significant difference was found between the antimicrobial effect of ethanol and two experimental medicaments. It indicates that ethanol, as the solvent of propolis, does not influence propolis’ antimicrobial effect. This finding concurs with others [45].

## Conclusions

Propolis is an effective agent against E. faeclis in the intracanal setting in our study, though its extraction is not yet standardised. It showed superiority over calcium hydroxide. The in vivo efficacy of propolis has yet to be investigated. Future studies of Propolis should standardize the process of extraction to obtain a uniform and consistent composition.
